# Components and classification of frailty and palliative care

**DOI:** 10.1016/j.tjfa.2026.100196

**Published:** 2026-09-05

**Authors:** Tjeerd van der Ploeg, Johan Wens, Robbert J J Gobbens

**Affiliations:** a Faculty of Health, Sports and Social Work, Inholland University of Applied Sciences, Amsterdam, the Netherlands; b Primary and Interdisciplinary Care Antwerp, Family Medicine and Population Health, University of Antwerp, Antwerp, Belgium; c Zonnehuisgroep Amstelland, Amstelveen, the Netherlands; d Tranzo, Tilburg University, Tilburg, the Netherlands

**Keywords:** Case study, Frailty, Palliative care needs, Classification, Regression analysis

## Abstract

**Background:**

Globally, the number of people aged 65 years and older is expected to double by 2050. With increasing age, the risk of frailty and the need for palliative care also rise. A shared understanding of how to assess frailty and determine the need for palliative care could support interprofessional collaboration in clinical practice. The transition from a frail condition to palliative care is a dynamic and individualized process. Although frailty and palliative care are distinct concepts, they may overlap. The present study aimed to examine the components of frailty and palliative care in community-dwelling older people. Early recognition by healthcare professionals of the components that contribute to severe frailty can enable the timely initiation of palliative care. Our second aim was to gain insight into whether an individual should be classified as frail or in need of palliative care according to healthcare professionals.

**Methods:**

We presented fifteen case studies to respondents who were selected based on their specific expertise in the Netherlands and Belgium. Each respondent was asked to identify, for each case study, the components that indicated frailty, palliative care needs, or both. Additionally, they were asked to rate their level of certainty regarding each indication. Wilcoxon tests were employed to assess differences, and regression analyses were conducted to identify the components most strongly associated with frailty and in need of palliative care.

**Results:**

In most cases, differences were observed between the identification of frailty and palliative care needs, with par-ticipants indicating that a patient could exhibit components of frailty without necessarily having palliative care needs (*p*-value *<* 0.05). In no case were palliative components recognized with greater certainty than components of frailty. The case in our study with the highest certainty score for frailty components showed a significant difference compared to the corresponding certainty score for palliative care needs. Conversely, the case with the highest certainty score for palliative care needs did not differ significantly from the certainty score for frailty components.

**Conclusions:**

Our study showed that healthcare professionals use different components to classify individuals as frail or in need of palliative care. In addition, these professionals were more certain in identifying frailty than in identifying palliative care needs, and may be less likely to recognize palliative care needs in their patients, who may already be quite frail. Improved understanding of the progression from frailty to the need for palliative care may support patient-centered care and increase enhance the quality of life for individuals requiring support.

## 1 Introduction

Globally, the number of people aged 65 years and older will double by 2050. With increasing age, the risk of multimorbidity, disability, and frailty also rises [[1], [2], [3]], thereby intensifying the need for palliative care. According to the Lancet Commission, approximately 40% of the 61 million people requiring palliative care are aged 70 or older [4].

In older people, healthcare professionals must pay attention to frailty because older people with frailty have a high risk of experiencing adverse outcomes. Well-known adverse outcomes are disability [5], an increase in healthcare utilization (e.g. hospitalization, institutionalization) [5,6], lower quality of life [6], and premature death [5,7].

Frequently used tools to capture the concept of frailty are the Phenotype of Frailty (PF), the Frailty Index (FI), and the Tilburg Frailty Indicator (TFI). The PF is characterized by a physical approach to frailty. Frailty is assessed using five criteria weakness, slowness, exhaustion, low physical activity, and unintentional weight loss [8]. The FI is developed by Mitnitski et al. (2001) [9] and contains many items, originally 92, including physical, psychological, and social limitations, and items referring to diseases and disability. The third instrument, the TFI, is based on an integral conceptual model of frailty [10], and includes in total fifteen components, referring to physical, psychological, and social frailty [11].

There are also many different definitions of palliative care. The World Health Organization defined palliative care as ‘an approach that improves the quality of life of patients and their families facing the problem associated with life-threatening illness, through the prevention and relief of suffering using early identification and impeccable assessment and treatment of pain and other problems, physical, psychosocial, and spiritual [12]. Palliative care includes end-of-life care and hospice transitions. It can be divided into primary palliative care, provided by healthcare professionals consisting of symptom man-agement as well as discussions based on advanced care planning, and specialty palliative care; this care is carried out by an interdisciplinary team with advanced training [13]. Much palliative care is organized and generally restricted to people with terminal diseases such as cancer or a disease for which it is also true that life expectancy will be limited [14], like amyotrophic lateral sclerosis (ALS).

However, initiating palliative care in older people who are severely frail is much less common, while these people often have an accumulation of problems in physical, psychological, and social functioning. Although palliative care is especially identified with cancer, the proportion of people with non-cancer (e.g. frailty) palliative care needs was significantly higher than in people with cancer palliative care needs [15]. In a cross-sectional study, a large variety of symptoms and complex needs experienced by hospitalized frail older people was identified; according to the research group, these people should be eligible for palliative care follow-up at home [16]. According to the Worldwide Hospice Palliative Care Alliance, palliative care should be interwoven into primary healthcare, which is cost-effective in providing more appropriate care at less cost because this can prevent unnecessary hospital admissions [17].

The associations between frailty and palliative care have been examined in several studies. For example, in a sample of intensive care unit survivors aged *≥*65 years, it was shown that frailty assessed with the PF could be a trigger for palliative care consultation [18]. Additionally, a systematic review and narrative synthesis focused on the end-of-life care needs of frail people showed that they had emotional distress and pain comparable to people with advanced cancer. Moreover, frail people have a range of physical (weakness) and psychosocial (anxiety) needs at the end of life that may benefit from palliative care [19]. Finally, Blanes-Selva et al. (2022) [20] proposed a machine-learning approach to predict frailty, assessed with a FI, in older people in supporting palliative care decision-making and concluded that a frailty assessment could be complementary for palliative care decision-making. Moreover, a recent study demonstrated that frailty, defined by the Hospital Frailty Risk Scores, had a significant impact on end-of-life health utilization (e.g. unplanned emergency room visits) among people with cancer [21].

According to Dewhurst et al. (2022) [22], a shared understanding of assessing frailty and the need for palliative care could support interprofessional collaboration in clinical practice. The transition from a frail condition to palliative care is a dynamic and individual process. Although frailty and palliative care are different concepts, they may overlap. The present study aimed to examine the components of frailty and palliative care in community-dwelling older people viewed from the perspective of primary healthcare professionals. Early recognition by healthcare professionals of components that cause someone to be severely frail can lead to palliative care being initiated promptly. Our second aim was to gain insight into whether an individual should be classified as frail or in need of palliative care according to healthcare professionals.

## 2 Methods

This study aimed to identify specific components of ‘frailty’ and ‘palliative care’ in the context of primary care. Fifteen case studies were presented. For each case, respondents assessed whether the described individual, based on the provided data, could be classified as ‘frail’ or as requiring ‘palliative care’, and indicated how certain they were about their classification. In addition, each participant was asked to indicate in each case those items that indicated frailty or palliative care needs or both. The items for the cases in our study were defined by two experts in the fields of frailty and palliative care. Prior to the study, members of the target population reviewed the instructions for clarity, completeness of background questions, and the ease of the scoring method. Feedback from seven healthcare professionals indicated no need for adjustments.

### 2.1 Respondents

Selecting respondents for a case study is a crucial process that strongly influences the quality and relevance of the research. In case studies, purposive sampling is a commonly used method, where respondents are selected based on their specific expertise to ensure the most relevant insights for the research objectives. In primary care, it is mainly general practitioners and nurses who come into frequent contact with frail people and those who need palliative care. Therefore we focused on the inclusion of these two disciplines in our study. Recruitment of respondents began in October 2023. The Dutch respondents were recruited via the Department of Geriatrics and Gerontology of VVN, the Dutch professional association for nurses and caregivers. Flemish participants were recruited through multiple professional networks, including the Flemish Organization of General Practitioners, the fifteen Flemish Palliative Care Networks, academic networks of general practitioner researchers affiliated with Flemish universities, and a dedicated network of home nurses. In this study, 63 respondents were involved in classifying the individuals in the fifteen cases.

### 2.2 Cases

The respondents were presented with fifteen cases. Each case contained several phrases (items) that could be used for classification as ‘frail’, ‘doubt’, or ‘palliative’. Additionally, the respondents were required to indicate, on a scale from 0 to 10 (higher score means more certain), how certain they were about the classification as either ‘frail’ or ‘palliative’.

### 2.3 Case merging

An expert in the field of frailty (expert A) and an expert in the field of palliative care (expert B) were asked to group the items that were involved in all cases. While expert A used the categories ‘determinant’, ‘physical’, ‘psychological’, ‘social’, and ‘adverse outcome’ for the grouping, expert B used the categories ‘biological’, ‘psychological’, ‘social’, and ‘spiritual’. The aim of this grouping was to compare the qualifications based on different standards of the two experts.

### 2.4 Analysis

The certainty scores for the classifications ‘frail’ and ‘palliative’ in a case were presented with the characteristics minimum and maximum, mean and median, standard deviation, and interquartile range. For each case, the certainty scores were compared using a paired Wilcoxon test. The frequencies of the items were presented per case as numbers, broken down by the classifications ‘frail’, ‘doubt’, and ‘palliative’. Univariable and multivariable analyses for the classification ‘frail’ versus ‘non-frail’ were performed using logistic regression, both per case and overall, based on the experts’ classifications [[23], [24], [25], [26]]. Items with a *p*-value *<*0.25 in the univariable analysis were included in the multivariable analysis. A *p*-value *<*0.05 was considered significant. All analyses were performed using R version 4.1.2 [27].

## 3 Results

The results section starts with the characteristics of the respondents (Table 1). Then, for the first case, a description follows, along with a frequency table showing the distribution of the items used for the classification ‘frail’, ‘doubt’, and ‘palliative’ (Table 2), following by a table presenting the certainty scores for these classifications (Table 3), and a table displaying the output of the logistic regression analysis for the classification of ‘frail’ versus ‘non-frail’ (Table 4).

**Table 1:** Characteristics respondents.

	n	%
**Country**		
the Netherlands	54	85.7
Flanders	9	14.3
**Gender**		
man	6	9.5
woman	56	88.9
I’d rather not say	1	1.6
**Age**		
25 to 34 years	5	8.1
35 to 44 years	15	24.2
45 to 54 years	15	24.2
55 to 64 years	22	35.5
65 to 74 years	5	8.1
**Position**		
other	11	17.5
GP	2	3.2
GP, CRA (Flanders)	1	1.6
GP, palliative care physician	2	3.2
nurse	42	66.7
nurse, other	5	7.9
**Experience**		
less than 5 years	11	18.0
5 to 10 years	10	16.4
10 to 20 years	15	24.6
20 to 30 years	16	26.2
30 to 40 years	6	9.8
40 years or more	3	4.9

GP=general practitioner.

CRA=coordinating and advisory physician in homes for the elderly.

**Table 2:** Distribution items.

	frail	doubt	palliative
14: had a partial nephrectomy	7	3	2
19: well-controlled type 2 diabetes	20	1	1
21: lives independently at home	2	0	0
24: daughter recently moved	8	0	0
28: city about 130 km away	3	0	0
30: lost 8 kg in the last 6 months (<10% of his body weight)	12	10	5
32: gets tired more quickly	10	4	7
4: 77 year old	5	0	0
5: widower	3	0	0
6: recently diagnosed	2	2	2
9: localized kidney cancer	10	8	9

**Table 3:** Certainty scores respondents.

	mean	SD	median	IQR	n
frail	6.8	2.2	7.0	3.0	32
palliative	5.1	3.0	5.0	5.0	32

Wilcoxon test *p*-value 0.018 SD=Standard Deviation IQR=Inter Quartile Range.

**Table 4:** Regression analysis case 1.

	Univariable		Multivariable	
	coefficient	*p*-value	coefficient	*p*-value
19 well controlled type 2 diabetes	2.127	0.005	0.580	0.513
30 lost 8 kg in the last 6 months 10 of his body weight	*−*0.808	0.064	*−*1.946	0.00
	**Univariable**		**Multivariable**	
	coefficient	*p*-value	coefficient	*p*-value
32 gets tired more quickly	*−*0.611	0.201	*−*1.818	0.005
6 recently diagnosed	*−*1.163	0.189	*−*2.416	0.015
9 localized kidney cancer	*−*1.196	0.007	*−*2.253	0.000
14 had a partial nephrectomy	*−*0.089	0.884		
32 gets tired more quickly	*−*0.611	0.201		
6 recently diagnosed	*−*1.163	0.189		
9 localized kidney cancer	*−*1.196	0.007		
14 had a partial nephrectomy	*−*0.089	0.884		
21 lives independently at home	15.173	0.988		
24 daughter recently moved	17.251	0.990		
28 city about 130 km away	15.186	0.986		
4 77 year old	16.211	0.988		
5 widower	15.186	0.986		

The results section continues with an overall table (Table 5), followed by a comparison of the grouping of the items by the two experts in the field of frailty and palliative care (Table 6). The results section ends with two summary tables containing the certainty scores (Table 7).

**Table 5:** Overall.

	n	%
frail	1231	65.5
doubt	450	24.0
palliative	197	10.5
	1878	100.0

**Table 6:** Expert A vs Expert B.

	Expert B				
	biological	psychological	social	spiritual	total
**Expert A**					
adverse outcome	204	20	78	8	310
determinant	834	56	128	15	1033
physical	317	9	73	0	399
psychological	0	99	0	15	114
social	0	14	8	0	22
total	1355	198	287	38	1878

**Table 7:** Summary certainty scores cases.

		mean		
case	n	frail	palliative	*p*-value*
1	32	6.8	5.1	0.018
2	29	8.2	7.0	0.015
3	25	8.0	7.3	0.418
4	22	8.4	6.6	0.006
5	18	4.6	2.3	0.006
6	20	7.1	4.7	0.005
7	18	7.9	4.2	0.001
8	16	8.1	4.1	0.001
9	15	6.6	5.4	0.220
10	14	8.1	2.9	0.001
11	16	8.2	7.1	0.073
12	14	8.1	6.9	0.261
13	17	7.8	6.7	0.265
14	15	8.1	5.0	0.004
15	13	6.9	6.1	0.191

*=*p*-value Wilcoxon test.

The first case will be discussed in detail and will serve as an example for interpreting the other fourteen cases in the appendices.

### 3.1 Characteristics respondents

Table 1 presents the numbers and percentages for the characteristics of the respondents. In total, 86% are from the Nether-lands, 89% are female, nearly 70% are aged 45 or older, approximately 75% are nurses, and about two-thirds have ten or more years of work experience.

### 3.2 Case 1

#### 3.2.1 Description

Marcus is a 77-year-old widower, recently diagnosed with localized kidney cancer. He will undergo a partial nephrectomy for this. He also has well-controlled type 2 diabetes. He lives independently at home, although his daughter recently moved to a town about 130 km away. He has lost 8 kg in the last 6 months (*<*10% of his body weight) and is getting tired more easily.

#### 3.2.2 Distribution items

Table 2 shows the distribution of items over the three classifications ‘frail’, ‘doubt’, and ‘palliative’ in counts. Each count represents the number of times an item was assigned by the respondents to one of the classifications ‘frail’, ‘doubt’, and ‘palliative’. For four items (30, 32, 6, and 9), the sum of the frequencies for ‘doubt’ and ‘palliative’ is higher than for ‘frail’ (15 versus 12 for item 30 e.g.). For the other items the frequency of ‘frail’ is higher than the sum of the frequencies for ‘doubt’ and ‘palliative’.

#### 3.2.3 Certainty scores

Table 3 presents the characteristics of the certainty scores for case 1. Of the respondents, 32 respondents indicated both scores. For the classifications ‘frail’ and ‘palliative’ the average scores are 6.8 and 5.1 respectively on a total score of 0 to 10. The difference in averages is significant (*p*-value 0.018, Wilcoxon).

#### 3.2.4 Uni- and multivariable analysis

Table 4 presents the output of the univariable and multivariable logistic regression analysis with ‘frail’ or ‘non-frail’ as the dependent variable. Items 19, 30, 32, 6, and 9 are included in the multivariable analysis because these items had *p*-values *<*0.25 in the univariable analysis. In the multivariable analysis, items 30, 32, 6 and 9 have *p*-values *<*0.05. In addition, these items also have negative coefficients, which indicates that for these items the probability of ‘frail’ is lower than the probability of ‘non-frail’.

### 3.3 Overall analyses

After combining all items of all cases, the total number of classified items by the respondents was 1878. Of the 1878 items, 65.5% were associated with ‘frailty’ and 10.5% with ‘palliative care’, while 24% of the items indicated ‘doubt’. Table 5 shows the overall classification by the respondents.

Table 6 shows the relation between the grouping of the items by expert A and by expert B. Of the 114 items grouped as ‘psychological’ by expert A, 99 were also grouped as ‘psychological’ by expert B. For the ‘social’ grouping by expert A, 22 items out of 22 corresponded with the ‘psychological’ or ‘social’ grouping by expert B. For the remaining grouping by expert A, the grouping mainly corresponded to the grouping ‘biological’ by expert B.

### 3.4 Summary tables

Table 7 summarizes the mean certainty scores for ‘frail’ and ‘palliative’ per case. The Wilcoxon test was used to analyze the differences and the resulting *p*-values are presented. For most cases, these *p*-values indicate significant differences in the interpretation of ‘frail’ versus ‘palliative’. Only for cases 3, 9, 11, 12, 13, and 15, the difference between ‘frail’ patients and ‘palliative’ patients was less clear for the respondents.

Table 8 is a summary table based on multivariate regression analysis for euch case that shows which items significantly indicate ‘frail’ and which items significantly indicate ‘non-frail’. The tables for each case are presented in detail in the appendices.

**Table 8:** Summary multivariable analyses cases.

	Frail	non-frail	coefficient	*p*-value
**case 1**				
	well controlled type 2 diabetes		0.580	0.513
		gets tired more quickly	−1.818	0.005
		lost 8 kg in the last 6 months 10 of his body weight	−1.946	0.002
		localized kidney cancer	−2.253	0.000
		recently diagnosed	−2.416	0.015
**case 2**				
	91 year old		2.138	0.046
	he has fallen several times in the last 6 months		1.942	0.014
	this has made him afraid to walk		1.445	0.033
	right hemispheric cerebral infarction		0.703	0.197
		symptoms were attributed to a combination of slow tumor progression	−2.398	0.000
		slowly progressive	−3.066	0.004
		local advanced rectal cancer	−3.066	0.000
**case 3**				
	Depressed		1.612	0.013
	type 2 diabetes		1.019	0.215
		as if he was giving up	−0.927	0.107
		dialysis restarted	−1.150	0.179
		severe graft versus host GVH disease	−1.397	0.010
		limited quality of life in the last 6 months	−1.486	0.012
**case 4**				
	difficulty adhering to her complex medication regimen		0.330	0.586
		obese	−1.210	0.013
		in the last 6 months she has lost more than 10 of her body weight 8 kilos	−1.711	0.003
		e GFR calculated as moderate stage 4 renal insufficiency 28 ml min 1	−2.657	0.000
	Frail	non-frail	coefficient	p-value
**case 5**				
		diagnosis of chronic kidney disease stage 4 e GFR 20 ml min 1	−1.910	0.007
**case 6**				
	requires considerable assistance with activities of daily living		1.023	0.342
		COPD	−1.972	0.001
		shortness of breath after walking about 50 m	−2.665	0.000
**case 7**				
		suffers from ischemic heart disease	−2.231	0.001
**case 8**				
	history of ulcerative colitis and polymyalgia rheumatica		0.746	0.491
		loss of weight and appetite	−1.403	0.033
		requires extensive nursing care from his family and home nurses	−1.403	0.033
		because of pain	−1.516	0.039
	Frail	non-frail	coefficient	p-value
**case 9**				
		develops edema on his legs during the day	−0.976	0.116
		due to heart failure	−1.564	0.010
**case 10**				
		becomes short of breath with moderate in	−1.191	0.058
		her second admission for the same problem in the last 10 months	−1.661	0.025
**case 11**				
	a fall that broke his nose		0.750	0.505
	needs help with clothes		0.617	0.586
		moderate dementia	−3.275	0.000
**case 12**				
	her dog died last week		1.872	0.082
	very upset		1.620	0.137
		moderately advanced Alzheimer s	−1.242	0.048
**case 13**				
	history of smoking for 40 pack years		1.536	0.163
	she cannot expect support from him		1.402	0.206
	tired after light housework		1.248	0.265
		ischemic heart disease and unstable angina pectoris	−0.949	0.116
		followed by severe COPD Gold D	−2.010	0.004
**case 14**				
		many comorbidities including peripheral vascular disease	−0.868	0.131
**case 15**				
		non invasive ventilation and intravenous antibiotics	−2.234	0.005
		third hospitalization in 9 months for exacerbations of her lung disease	−2.303	0.001

## 4 Discussion

Based on fifteen standardized cases, professional healthcare providers (doctors and nurses) in the Netherlands and Belgium were asked to make their assessment of components of frailty and palliative needs in the proposed cases.

In most cases, differences were noted between the picture of frailty and palliative needs, where it was indicated that a patient could have components of frailty without having palliative needs (*p*-value *<*0.05). In no case could palliative features be recognized with more certainty than features of frailty. Healthcare providers may be less likely to recognize or define palliative care needs in their patients, who may already be quite frail. This might have consequences in the palliative care delivery. These findings fit in nicely with the results of a systematic review from 2019 on the needs of people with frailty at the end of life [28]. Here, evidence is highlighted that people with frailty experience pain and emotional distress at levels similar to people with cancer and also report a range of physical and psychosocial needs, including weakness and anxiety that may benefit from palliative care. A cross-sectional study by Hentsch et al. (2023) [29] concludes as well that older, frail, housebound patients have specific needs that differ from non-frail patients and should guide future palliative care provision. How and when palliative care should be provided to this population remains to be determined. Hofstede et al. (2016) [30] concludes their comparative study with the fact that compared with bereaved relatives of patients with cancer, bereaved relatives of patients with organ failure or frailty were more likely to negatively assess the palliative care provided to both the patient and themselves.

The case in our study with the highest certainty score on frailty components (case 4) has a significant difference with the certainty score on palliative care needs, while the case with the highest certainty score on palliative care needs (case 3) does not differ significantly from the certainty on frailty components. This might illustrate as well that once palliative care needs implicit frailty is recognized as well, but not vice versa. An earlier recognition of palliative care needs in people with components of frailty can initiate an earlier palliative care trajectory with more emphasis on quality of life. Amblas-Novelals et al. (2021) [31] also show that it may be useful to include the degree of frailty in the evaluation of palliative care needs. The main components to differ significantly (*p*-value *<*0.05) between frailty and the need for palliative care in our study are age (*>*90 years old), frequent falls, and fear of falling, in addition to depressive symptoms. It may seem remarkable that ‘old age’ is seen as a specific feature of frailty, and is not more strongly focused on palliative care. More knowledge about frailty progression in the years before death can guide effective palliative care integration to enhance relationships between frail individuals, informal caregivers, and healthcare professionals while promoting patient-centered care and health-related suffering will be more intentionally relieved [32].

### 4.1 Strengths and limitations

The strength of our study lies mainly in the clinically relevant approach in which healthcare professionals were invited to share their views on end-of-life care using standardized clinical cases that closely reflect daily practice. In addition, the opinions of both doctors and nurses were sought, both in Flanders and in the Netherlands. However, perhaps due to the amount of time required to complete these fifteen cases and any lack of interest in the topics as frailty and palliative care in the primary care setting, not many fully completed surveys could be analyzed.

An important consequence of this is that a logical regression could be calculated with frailty as a reference, but not with the need for palliative care as a reference, which could perhaps have provided further clarity in the demarcation of the two concepts.

We used fifteen cases. For each case, the 63 reviewers could use predefined keywords that could be associated with the classifications ‘frail’, ‘doubt’, and ‘palliative’. For a multivariate regression analysis, the numbers are somewhat low and the output should therefore be considered indicative. The respondents were from the Netherlands and Belgium. In both the Netherlands and in Belgium, general practitioners and nurses play an important role in primary palliative care and that their training prepares them for this role.

In the Netherlands, the PaTz (Palliatice care at home) method has been developed. This method is perceived to improve coordination, continuity and communication in palliative care in the Netherlands. A cross-sectional study showed that general practitioners and community nurses found PaTz beneficial for knowledge collaboration, coordination and continuity of care [33].

In Belgium, also specific attention was paid to the implementation of care pathways for palliative care in primary care. A study of Leysen (2025) [34] demonstrated that an integrated care pathway for primary palliative care helps to identify symp-toms early and assess the patients wishes, although factors at both the macro and micro levels can influence implementation.

A follow-up study with more cases and more respondents from other countries would improve generalizability.

## 5 Conclusion

Our study showed that healthcare professionals use other components to designate individuals as frail or in need of palliative care. In addition, these professionals are more certain that someone is frail than that someone needs palliative care. They may be less likely to recognize palliative care needs in their patients, who may already be quite frail. More knowledge among healthcare professionals about frailty progression in relation to starting palliative care may support patient-centered care and increase the quality of life of the individual in need of care.

## Funding

This research received no external funding.

## Ethical aspects

For this study, ethical review and approval in accordance with medical ethical procedures in the Netherlands was not required.

## Informed consent statement

Not applicable.

## Declaration of the use of AI

The authors did not use generative AI or AI-assisted technologies in the preparation of this manuscript.

## CRediT authorship contribution statement

**Tjeerd van der Ploeg:** Writing – review & editing, Software, Methodology, Formal analysis, Data curation. **Johan Wens:** Writing – review & editing, Supervision, Data curation, Conceptualization. **Robbert J J Gobbens:** Writing – review & editing, Writing – original draft, Supervision, Methodology, Conceptualization.

## Declaration of competing interest

All authors declare that they have no financial conflict of interest.

## Figures and Tables

**Table 9:** Distribution items.

	frail	doubt	palliative
11: slowly progressive	1	5	8
16: right hemispheric cerebral infarction	20	4	2
18: 5 years before the cancer was diagnosed	0	2	2
20: lives in a nursing home	6	0	0
23: wife died 3 years ago	6	0	0
26: can go to the toilet independently	1	0	0
29: he has fallen several times in the last 6 months	23	2	0
3: 91 year old	14	1	0
33: without major consequences	0	1	0
37: this has made him afraid to walk	21	2	1
42: good appetite	1	0	1
45: weight is relatively stable	1	1	0
48: suffers a lot from abdominal cramps	3	2	1
5: widower	8	0	0
	frail	doubt	palliative
52: in the hospital 3 times in the last 6 months	7	6	2
56: tests did not provide a diagnosis	0	1	1
60: symptoms were attributed to a combination of slow tumor progression	3	17	3
9: local advanced rectal cancer	2	14	12

**Table 10:** Certainty scores respondents.

	mean	SD	median	IQR	n
frail	8.2	1.8	9.0	2.0	29
palliative	7.0	2.3	7.0	3.0	29

Wilcoxon test *p*-value 0.015 SD=Standard Deviation IQR=Inter Quartile Range.

**Table 11:** Regression analysis case 2.

	Univariable		Multivariable	
	coefficient	*p*-value	coefficient	*p*-value
11 slowly progressive	*−*2.962	0.005	*−*3.066	0.004
16 right hemispheric cerebral infarction	1.072	0.028	0.703	0.197
29 he has fallen several times in the last 6 months	2.388	0.001	1.942	0.014
3 91 year old	2.504	0.017	2.138	0.046
37 this has made him afraid to walk	1.859	0.003	1.445	0.033
60 symptoms were attributed to a combination of slow tumor progression	*−*2.371	0.000	*−*2.398	0.000
9 local advanced rectal cancer	*−*3.135	0.000	*−*3.066	0.000
23 wife died 3 years ago	16.367	0.987		
26 can go to the toilet independently	14.323	0.987		
18 5 years before the cancer was diagnosed	*−*15.862	0.983		
20 lives in a nursing home	16.367	0.987		
33 without major consequences	*−*14.828	0.987		
5 widower	16.386	0.985		
42 good appetite	*−*0.254	0.858		
45 wt is relatively stable	*−*0.254	0.858		
48 suffers a lot from abdominal cramps	*−*0.259	0.755		
52 in the hospital 3 times in the last 6 months	*−*0.415	0.440		
56 tests did not provide a diagnosis	*−*15.840	0.988		

**Table 12:** Distribution items.

	frail	doubt	palliative
11: type 2 diabetes	7	1	1
15: hypertension	10	9	5
22: dialysis restarted	2	2	3
25: severe graft versus host (GVH) disease	5	9	7
3: unmarried male	2	0	0
30: limited quality of life in the last 6 months	4	10	4
33: sharp decline in physical capacity	11	7	3
37: unable to move without assistance	8	5	1
39: mother died a year and a half ago	7	0	0
44: depressed	19	3	0
49: as if he was giving up	5	6	4
52: low adherence to therapy	13	6	2
57: lost interest in his self-care and his own goals	11	6	2
58: He was recently admitted to the hospital	3	3	1
	frail	doubt	palliative
59: to give his immediate environment some rest	1	0	1
8: first kidney transplant 10 years ago	6	5	1

**Table 13:** Certainty scores respondents.

	mean	SD	median	IQR	n
Frail	8.0	2.5	9.0	2.0	25
palliative	7.3	2.3	8.0	2.0	25

Wilcoxon test *p*-value 0.418 SD=Standard Deviation IQR=Inter Quartile Range.

**Table 14:** Regression analysis case 3.

	Univariable		Multivariable	
	coefficient	*p*-value	coefficient	*p*-value
11 type 2 diabetes	1.234	0.129	1.019	0.215
22 dialysis restarted	*−*1.010	0.234	*−*1.150	0.179
	**Univariable**		**Multivariable**	
	coefficient	*p*-value	coefficient	*p*-value
25 severe graft versus host GVH disease	*−*1.344	0.011	*−*1.397	0.010
30 limited quality of life in the last 6 months	*−*1.421	0.015	*−*1.486	0.012
44 depressed	1.936	0.002	1.612	0.013
49 as if he was giving up	*−*0.810	0.152	*−*0.927	0.107
3 unmarried male	15.520	0.988		
15 hypertension	*−*0.448	0.306		
33 sharp decline in physical capacity	0.035	0.939		
37 unable to move without assistance	0.239	0.668		
39 mother died a year and a half ago	16.566	0.985		
58 He was recently admitted to the hospital	*−*0.362	0.640		
59 to give his immediate environment some rest	*−*0.064	0.964		
52 low adherence to therapy	0.466	0.323		
57 lost interest in his self care and his own goals	0.279	0.566		
8 first kidney transplant 10 years ago	*−*0.067	0.910		

**Table 15:** Distribution items.

	frail	doubt	palliative
11: they live in a small house	1	0	0
14: no longer living there satisfactorily	8	0	0
17: very noisy	7	0	0
19: have no contact with neighbors or other residents	13	0	0
23: have difficulty making ends meet on their income	18	0	0
27: familial polycystic kidney disease	10	5	6
3: 66 years old	1	0	0
31: obese	9	9	3
34: almost completely bedridden	9	6	3
37: difficulty adhering to her complex medication regimen	14	4	0
44: e-GFR calculated as moderate (stage 4) renal insufficiency (28 ml/min/1)	3	12	5
50: in the last 6 months she has lost more than 10% of her body weight (8 kilos)	5	7	4
57: exhausted	5	2	2
	frail	doubt	palliative
62: completely dependent for most activities of daily living	6	6	1

**Table 16:** Certainty scores respondents.

	mean	SD	median	IQR	n
frail	8.4	1.6	9.0	2.5	22
palliative	6.6	2.1	7.0	3.0	22

Wilcoxon test *p*-value 0.006 SD=Standard Deviation IQR=Inter Quartile Range.

**Table 17:** Regression analysis case 4.

	Univariable		Multivariable	
	coefficient	*p*-value	coefficient	*p*-value
31 obese	*−*0.750	0.110	*−*1.210	0.013
37 difficulty adhering to her complex medication regimen	0.962	0.102	0.330	0.586
44 e GFR calculated as moderate stage 4 renal insufficiency 28 ml min 1	*−*2.338	0.000	*−*2.657	0.000
	**Univariable**		**Multivariable**	
	coefficient	*p*-value	coefficient	*p*-value
50 in the last 6 months she has lost more than 10 of her body weight 8 kilos	*−*1.274	0.023	*−*1.711	0.003
11 they live in a small house	14.201	0.987		
14 no longer living there satisfactorily	16.268	0.985		
17 very noisy	16.259	0.986		
19 have no contact with neighbors or other residents	17.319	0.987		
23 have difficulty making ends meet on their income	17.373	0.985		
27 familial polycystic kidney disease	*−*0.532	0.253		
3 66 years old	14.201	0.987		
34 almost completely bedridden	*−*0.416	0.403		
62 completely dependent for most activities of daily living	*−*0.569	0.324		
57 exhausted	*−*0.159	0.818		

**Table 18:** Distribution items.

	frail	doubt	palliative
12: hypertension	3	0	1
14: currently being treated with blood pressure medication	4	0	0
16: has had 2 hospitalizations in the last year	3	0	0
21: pyelonephritis	6	2	1
28: traffic accident that resulted in a broken leg	3	0	0
3: 65 years old	1	0	0
32: fully recovered	1	0	0
38: good appetite	1	0	0
4: divorced	1	0	0
40: her weight is stable	2	0	0
43: recently discharged	3	0	0
50: house for sale	1	0	0
54: looking for work	1	0	0
8: diagnosis of chronic kidney disease stage 4 (e-GFR = 20ml/min/1)	10	5	4

**Table 19:** Certainty scores respondents.

	mean	SD	median	IQR	n
frail	4.6	2.7	4.5	3.2	20
palliative	2.3	2.5	1.0	2.0	18

Wilcoxon test *p*-value 0.006 SD=Standard Deviation IQR=Inter Quartile Range.

**Table 20:** Regression analysis case 5.

	Univariable		Multivariable	
	coefficient	*p*-value	coefficient	*p*-value
8 diagnosis of chronic kidney disease stage 4 e GFR 20 ml min 1	*−*1.910	0.007	*−*1.91	0.007
12 hypertension	*−*0.027	0.982		
14 currently being treated with blood pressure medication	16.547	0.993		
16 has had 2 hospitalizations in the last year	16.520	0.994		
21 pyelonephritis	*−*0.531	0.504		
28 traffic accident that resulted in a broken leg	16.520	0.994		
3 65 years old	15.467	0.995		
32 fully recovered	15.467	0.995		
	**Univariable**		**Multivariable**	
	coefficient	*p*-value	coefficient	*p*-value
38 good appetite	15.467	0.995		
4 divorced	15.467	0.995		
40 her weight is stable	15.493	0.993		
43 recently discharged	16.520	0.994		
50 house for sale	15.467	0.995		
54 looking for work	15.467	0.995		

**Table 21:** Distribution items.

	frail	doubt	palliative
16: he bit Alfred on his right hand	1	1	0
18: did not seek medical advice	6	0	0
21: wound became infected and painful	7	1	0
23: resulted in admission to intensive care for septic shock	9	2	1
3: 76 years old	4	0	0
31: slow to recover	5	1	0
35: bedridden for most of the day	13	4	0
38: requires considerable assistance with activities of daily living	12	1	0
39: Alfred has lost 4 kg in body weight in the last 6 months	11	2	0
4: single	4	0	0
42: heavy pipe smoker for at least 40 years	6	3	1
45: COPD	6	6	4
48: shortness of breath after walking about 50 m	3	7	3
6: lived in a remote valley his entire life	3	0	0

**Table 22:** Certainty scores respondents.

	mean	SD	median	IQR	n
frail	7.1	1.9	7.0	1.0	21
palliative	4.7	3.0	4.5	5.0	20

Wilcoxon test *p*-value 0.005 SD=Standard Deviation IQR=Inter Quartile Range.

**Table 23:** Regression analysis case 6.

	Univariable		Multivariable	
	coefficient	*p*-value	coefficient	*p*-value
38 requires considerable assistance with activities of daily living	1.712	0.106	1.023	0.342
45 COPD	*−*1.646	0.003	*−*1.972	0.001
48 shortness of breath after walking about 50 m	*−*2.374	0.001	*−*2.665	0.000
16 he bit Alfred on his right hand	*−*0.905	0.526		
18 did not seek medical advice	15.746	0.987		
21 wound became infected and painful	1.111	0.307		
23 resulted in admission to intensive care for septic shock	0.231	0.741		
3 76 years old	15.723	0.990		
	**Univariable**		**Multivariable**	
	coefficient	*p*-value	coefficient	*p*-value
31 slow to recover	0.750	0.500		
35 bedridden for most of the day	0.331	0.586		
39 Alfred has lost 4 kg in body weight in the last 6 months	0.891	0.263		
4 single	15.723	0.990		
42 heavy pipe smoker for at least 40 years	*−*0.529	0.435		
6 lived in a remote valley his entire life	15.711	0.991		

**Table 24:** Distribution items.

	frail	doubt	palliative
11: hospitalized twice in the last year	10	1	0
15: pneumonia	9	0	0
22: fall	4	0	0
29: has family help with cooking	8	0	0
3: 86 years old	7	1	0
31: uses a walker to get around	7	0	0
33: remains in bed or in a chair for most of the day	15	3	0
35: daughter visits her every Tuesday	1	0	0
7: Surinamese origin	3	0	0
9: suffers from ischemic heart disease	11	6	2

**Table 25:** Certainty scores respondents.

	Mean	SD	median	IQR	n
Frail	7.9	1.4	8.0	2.0	20
palliative	4.2	2.8	3.5	3.0	18

Wilcoxon test *p*-value *<*0.001 SD=Standard Deviation IQR=Inter Quartile Range.

**Table 26:** Regression analysis case 7.

	Univariable		Multivariable	
	coefficient	*p*-value	coefficient	*p*-value
9 suffers from ischemic heart disease	*−*2.231	0.001	*−*2.231	0.001
11 hospitalized twice in the last year	0.613	0.575		
15 pneumonia	16.941	0.994		
22 fall	15.868	0.994		
29 has family help with cooking	15.926	0.991		
3 86 years old	0.211	0.850		
31 uses a walker to get around	15.912	0.992		
33 remains in bed or in a chair for most of the day	*−*0.182	0.800		
35 daughter visits her every Tuesday	13.827	0.992		
7 Surinamese origin	14.854	0.991		

**Table 27:** Distribution items.

	frail	doubt	palliative
10: feels lonely	14	0	0
12: has moderate heart failure	9	3	1
17: history of ulcerative colitis and polymyalgia rheumatica	12	1	0
20: requires high doses of steroids	2	1	0
24: to symptoms of diabetes	6	1	0
28: loss of weight and appetite	7	5	0
30: lost 8 kg (>10% of his body weight) in the last 6 months	10	2	0
33: appetite has improved with starting a PPI	1	0	0
37: spends most of his time in bed	12	5	0
39: because of pain	5	4	0
43: requires extensive nursing care from his family and home nurses	7	4	1
44: he also has help with the housework	7	0	0
6: widower for two years	7	0	0
8: misses his wife daily	6	0	0

**Table 28:** Certainty scores respondents.

	mean	SD	median	IQR	n
Frail	8.1	1.4	8.0	2.0	18
Palliative	4.1	2.3	4.5	3.2	16

Wilcoxon test *p*-value 0.001 SD=Standard Deviation IQR=Inter Quartile Range.

**Table 29:** Regression analysis case 8.

	Univariable		Multivariable	
	coefficient	*p*-value	coefficient	*p*-value
17 history of ulcerative colitis and polymyalgia rheumatica	1.248	0.241	0.746	0.491
28 loss of weight and appetite	*−*1.113	0.077	*−*1.403	0.033
39 because of pain	*−*1.204	0.089	*−*1.516	0.039
43 requires extensive nursing care from his family and home nurses	*−*1.113	0.077	*−*1.403	0.033
10 feels lonely	16.387	0.988		
12 has moderate heart failure	*−*0.575	0.371		
30 lost 8 kg 10 of his body weight in the last 6 months	0.314	0.697		
20 requires high doses of steroids	*−*0.646	0.604		
	**Univariable**		**Multivariable**	
	coefficient	*p*-value	coefficient	*p*-value
24 to symptoms of diabetes	0.492	0.655		
8 misses his wife daily	15.303	0.988		
33 appetite has improved with starting a PPI	14.254	0.992		
37 spends most of his time in bed	*−*0.522	0.369		
44 he also has help with the housework	16.313	0.991		
6 widower for two years	16.313	0.991		

**Table 30:** Distribution items.

	frail	doubt	palliative
17: stately mansion	1	0	0
27: diagnosis of left ventricular insufficiency	9	4	3
32: acute heart failure 20 years ago	5	1	1
36: Parkinson’s	8	4	3
4: Turkish	2	0	0
40: walking more difficult	7	0	0
46: less and less strength in his hands	9	0	0
5: 89-year-old man	4	1	0
51: admitted to hospital twice in the last 6 months	6	2	2
52: due to heart failure	5	6	3
55: shortness of breath with mild exertion	7	4	1
57: develops edema on his legs during the day	6	4	2
	frail	doubt	palliative
62: move to a service flat with his wife	5	0	0
72: difficult for them to give up their home	4	0	0
81: can take care of himself	1	0	0
83: helps his wife with activities of daily living	1	0	0

**Table 31:** Certainty scores respondents.

	mean	SD	median	IQR	n
frail	6.6	1.9	7.0	3.0	17
palliative	5.4	3.4	5.0	6.0	15

Wilcoxon test *p*-value 0.220 SD=Standard Deviation IQR=Inter Quartile Range.

**Table 32:** Regression analysis case 9.

	Univariable		Multivariable	
	coefficient	*p*-value	coefficient	*p*-value
52 due to heart failure	*−*1.440	0.016	*−*1.564	0.010
57 develops edema on his legs during the day	*−*0.749	0.222	*−*0.976	0.116
17 stately mansion	14.910	0.992		
27 diagnosis of left ventricular insufficiency	*−*0.485	0.374		
32 acute heart failure 20 years ago	0.262	0.760		
36 Parkinson s	*−*0.617	0.269		
4 Turkish	14.923	0.988		
40 walking more difficult	16.989	0.991		
46 less and less strength in his hands	17.017	0.990		
5 89 year old man	0.744	0.512		
51 admitted to hospital twice in the last 6 months	*−*0.288	0.671		
72 difficult for them to give up their home	15.949	0.989		
55 shortness of breath with mild exertion	*−*0.370	0.550		
83 helps his wife with activities of daily living	14.910	0.992		
62 move to a service flat with his wife	15.962	0.988		
81 can take care of himself	14.910	0.992		

**Table 33:** Distribution items.

	frail	doubt	palliative
12: spent a large part of her life in poverty	11	1	0
14: caused stress	9	1	0
16: drank alcohol excessively	12	2	0
18: smoked an average of a pack of cigarettes per day	11	2	0
22: hospitalized	1	0	0
25: mild heart failure episode	9	1	2
3: 88 years old	5	1	0
30: becomes short of breath with moderate in	10	3	2
33: her second admission for the same problem in the last 10 months	5	3	1
35: lives in a nursing home	3	0	0
4: at age 80, she is divorced	5	0	0
43: spent most of her time in a chair or bed	9	2	0
53: able to move to bathroom with help from nursing staff	4	0	0

**Table 34:** Certainty scores respondents.

	mean	SD	median	IQR	n
frail	8.1	1.2	8.0	2.0	17
palliative	2.9	2.6	2.0	3.5	14

Wilcoxon test *p*-value 0.001 SD=Standard Deviation IQR=Inter Quartile Range.

**Table 35:** Regression analysis case 10.

	Univariable		Multivariable	
	coefficient	*p*-value	coefficient	*p*-value
30 becomes short of breath with moderate in	*−*0.965	0.115	*−*1.191	0.058
33 her second admission for the same problem in the last 10 months	*−*1.432	0.047	*−*1.661	0.025
12 spent a large part of her life in poverty	0.975	0.364		
14 caused stress	0.750	0.488		
16 drank alcohol excessively	0.329	0.682		
18 smoked an average of a pack of cigarettes per day	0.230	0.776		
22 hospitalized	14.078	0.992		
25 mild heart failure episode	*−*0.454	0.526		
	**Univariable**		**Multivariable**	
	coefficient	*p*-value	coefficient	*p*-value
3 88 years old	0.117	0.917		
43 spent most of her time in a chair or bed	0.006	0.994		
53 able to move to bathroom with help from nursing staff	15.111	0.990		
35 lives in a nursing home	15.100	0.991		
4 at age 80 she is divorced	16.122	0.993		

**Table 36:** Distribution items.

	frail	doubt	palliative
10: is his primary caregiver	0	1	0
12: he has been hospitalized 3 times in the last 9 months	11	3	1
18: urinary tract infection	5	1	1
24: pneumonia	6	1	1
30: a fall that broke his nose	8	1	0
4: 80 years old	4	0	0
45: needs help with clothes	7	1	0
53: sometimes needs help with personal care	8	0	0
7: moderate dementia	2	11	3

**Table 37:** Certainty scores respondents.

	mean	SD	median	IQR	n
frail	8.2	1.8	9.0	3.0	17
palliative	7.1	3.0	8.0	5.0	16

Wilcoxon test *p*-value 0.073 SD=Standard Deviation IQR=Inter Quartile Range.

**Table 38:** Regression analysis case 11.

	Univariable		Multivariable	
	coefficient	*p*-value	coefficient	*p*-value
30 a fall that broke his nose	1.496	0.170	0.750	0.505
45 needs help with clothes	1.340	0.223	0.617	0.586
7 moderate dementia	*−*3.440	0.000	*−*3.275	0.000
10 is his primary caregiver	*−*16.320	0.991		
12 he has been hospitalized 3 times in the last 9 months	0.367	0.568		
18 urinary tract infection	0.223	0.799		
24 pneumonia	0.427	0.617		
4 80 years old	15.935	0.989		
53 sometimes needs help with personal care	17.024	0.990		

**Table 39:** Distribution items.

	frail	doubt	palliative
10: resident of an assisted living facility	3	0	0
28: she fractured her 6th cervical vertebra	6	6	0
3: divorced woman	4	0	0
30: x-ray shows an unstable fracture	4	3	1
32: has not been mobile since the fall	6	5	2
34: needs help transferring from bed to chair	5	3	0
37: requires quite a bit of help with her care needs	5	4	0
45: blood transfusions are not allowed because of her religious beliefs	5	3	3
48: lost her appetite over the last few months	8	3	2
5: 77 years	2	0	0
54: lost 2 pounds of her body weight in the last month	6	2	2
56: her dog died last week	9	1	0
61: very upset	7	1	0
7: moderately advanced Alzheimer’s	4	8	2

**Table 40:** Certainty scores respondents.

	mean	SD	median	IQR	n
frail	8.1	1.9	8.0	1.0	17
palliative	6.9	2.6	8.0	4.2	14

Wilcoxon test *p*-value 0.261 SD=Standard Deviation IQR=Inter Quartile Range.

**Table 41:** Regression analysis case 12.

	Univariable		Multivariable	
	coefficient	*p*-value	coefficient	*p*-value
56 her dog died last week	1.935	0.071	1.872	0.082
61 very upset	1.653	0.128	1.620	0.137
7 moderately advanced Alzheimer s	*−*1.451	0.020	*−*1.242	0.048
10 resident of an assisted living facility	16.235	0.991		
28 she fractured her 6th cervical vertebra	*−*0.413	0.497		
3 divorced woman	16.249	0.989		
30 x ray shows an unstable fracture	*−*0.398	0.586		
32 has not been mobile since the fall	*−*0.589	0.317		
	**Univariable**		**Multivariable**	
	coefficient	*p*-value	coefficient	*p*-value
34 needs help transferring from bed to chair	0.148	0.844		
37 requires quite a bit of help with her care needs	*−*0.161	0.818		
45 blood transfusions are not allowed because of her religious beliefs	*−*0.610	0.337		
48 lost her appetite over the last few months	0.109	0.856		
5 77 years	15.221	0.988		
54 lost 2 pounds of her body weight in the last month	0.036	0.957		

**Table 42:** Distribution items.

	frail	doubt	palliative
10: followed by severe COPD (Gold D)	3	9	4
13: shortness of breath with light activities such as walking 100 m on level ground	6	3	2
18: admitted to hospital for pneumonia	5	4	0
21: lost 10 kg	6	2	2
27: never recovered to her previous level	4	3	1
32: tired after light housework	6	0	1
36: ischemic heart disease and unstable angina pectoris	6	7	2
40: sometimes needs help from her husband	4	1	0
42: family lives abroad	6	0	0
43: she cannot expect support from him	7	1	0
8: history of smoking for 40 pack years	8	1	0

**Table 43:** Certainty scores respondents.

	mean	SD	median	IQR	n
frail	7.8	2.0	8.0	2.0	17
palliative	6.7	2.6	7.0	4.0	17

Wilcoxon test *p*-value 0.265 SD=Standard Deviation IQR=Inter Quartile Range.

**Table 44:** Regression analysis case 13.

	Univariable		Multivariable	
	coefficient	p-value	coefficient	p-value
10 followed by severe COPD Gold D	−2.126	0.002	−2.010	0.004
32 tired after light housework	1.522	0.166	1.248	0.265
36 ischemic heart disease and unstable angina pectoris	−0.886	0.120	−0.949	0.116
43 she cannot expect support from him	1.695	0.120	1.402	0.206
8 history of smoking for 40 pack years	1.847	0.087	1.536	0.163
27 never recovered to her previous level	−0.379	0.607		
13 shortness of breath with light activities such as walking 100 m on level ground	−0.187	0.770		
18 admitted to hospital for pneumonia	−0.139	0.844		
21 lost 10 kg	0.062	0.928		
42 family lives abroad	16.320	0.987		
	**Univariable**		**Multivariable**	
	coefficient	p-value	coefficient	p-value
40 sometimes needs help from her husband	1.081	0.342		

**Table 45:** Distribution items.

	frail	doubt	palliative
12: poor appetite	7	3	1
14: has lost >10% of his body weight in the last six months (5 kilos)	9	5	1
17: almost completely bedridden	8	6	1
19: is capable of limited self-care	7	2	0
2: 80 years	3	0	0
21: lives in a residential care center	2	0	0
4: single	5	0	0
5: suffers from moderate COPD	9	5	0
8: many comorbidities including peripheral vascular disease	7	6	2

**Table 46:** Certainty scores respondents.

	mean	SD	median	IQR	n
frail	8.1	1.8	8.0	2.0	17
palliative	5.0	3.2	4.0	4.0	15

Wilcoxon test *p*-value 0.004 SD=Standard Deviation IQR=Inter Quartile Range.

**Table 47:** Regression analysis case 14.

	Univariable		Multivariable	
	coefficient	*p*-value	coefficient	*p*-value
8 many comorbidities including peripheral vascular disease	*−*0.868	0.131	*−*0.868	0.131
12 poor appetite	*−*0.020	0.976		
14 has lost 10 of his body weight in the last six months 5 kilos	*−*0.208	0.721		
17 almost completely bedridden	*−*0.539	0.346		
19 is capable of limited self care	0.742	0.374		
2 80 years	16.043	0.991		
21 lives in a residential care center	16.024	0.992		
4 single	16.081	0.988		
5 suffers from moderate COPD	0.012	0.984		

**Table 48:** Distribution items.

	frail	doubt	palliative
12: third hospitalization in 9 months for exacerbations of her lung disease	7	3	3
19: non-invasive ventilation and intravenous antibiotics	5	3	1
28: her husband Jim admitted with a stroke	6	0	0
31: died suddenly	10	0	0
35: depressed	14	0	0
37: anxious	13	0	0
4: 72-year-old woman	1	0	0
40: worried she can’t cope without Jim	7	1	0
59: can walk to local store which is less than 200 m from her home	1	0	0
62: has to stop halfway to catch her breath	7	1	1
66: daughter lives nearby to help with housework and support her	1	0	0
7: diagnosed with moderate COPD	10	3	0

**Table 49:** Certainty scores respondents.

	mean	SD	median	IQR	n
frail	6.9	1.8	7.0	3.2	16
palliative	6.1	2.9	6.0	4.0	13

Wilcoxon test *p*-value 0.191 SD=Standard Deviation IQR=Inter Quartile Range.

**Table 50:** Regression analysis case 15.

	Univariable		Multivariable	
	coefficient	*p*-value	coefficient	*p*-value
12 third hospitalization in 9 months for exacerbations of her lung disease	*−*1.861	0.004	*−*2.303	0.001
19 non invasive ventilation and intravenous antibiotics	*−*1.636	0.027	*−*2.234	0.005
35 depressed	17.119	0.992		
37 anxious	17.105	0.992		
28 her husband Jim admitted with a stroke	16.008	0.992		
31 died suddenly	16.062	0.990		
59 can walk to local store which is less than 200 m from her home	13.944	0.992		
62 has to stop halfway to catch her breath	*−*0.426	0.618		
4 72 year old woman	13.944	0.992		
40 worried she can t cope without Jim	0.336	0.761		
	**Univariable**		**Multivariable**	
	coefficient	*p*-value	coefficient	*p*-value
66 daughter lives nearby to help with housework and support her	13.944	0.992		
7 diagnosed with moderate COPD	*−*0.508	0.483		

## Data Availability

The data presented in this study are available upon request from the corresponding author.

## Appendices A Case 2

### A.1 Description

John is 91 years old and a widower. He has a locally advanced rectal cancer that is slowly progressing. He had a right hemispheric stroke 10 years ago, 5 years before the cancer was diagnosed. He has been living in a residential care center since his wife died 3 years ago. He can go to the toilet independently, but he has fallen several times in the last 6 months. Fortunately, this was without major consequences. However, this has made him afraid to walk. John still has a good appetite and his weight is relatively stable. He suffers a lot from abdominal cramps, for which he has been admitted to the hospital 3 times in the last 6 months. The tests did not provide a diagnosis and his symptoms were attributed to a combination of slow tumor progression, constipation, and intestinal hypoperfusion.

## B Case 3

### B.1 Description

Richard, a 55-year-old single man, received his first kidney transplant 10 years ago for type 2 diabetes. He also has hypertension, ischemic heart disease, chronic obstructive pulmonary disease (COPD), and obesity. At age 55, dialysis was restarted for severe Graft versus Host (GVH) disease. He reports a very limited quality of life in the last 6 months, with a sharp decline in physical capacity. He is now unable to move around unassisted. His mother died a year and a half ago, and since then he has become depressed and feels like he is ‘giving up´. He is not very compliant with his medication regimen and has lost interest in his self-care and personal goals. He was recently admitted to the hospital to give his immediate environment some rest.

## C Case 4

### C.1 Description

Pauline is 66 years old and is married to Cees who is 70 years old. They live in a small house in the center of a city. They are no longer satisfied with their living there; there is a lot of noise pollution and they have no contact with the neighbors or other residents. Pauline and Cees have difficulty making ends meet with their income. Pauline suffers from familial polycystic kidney disease. In addition, she is obese and has type 2 diabetes, hypertension, COPD with 30 pack years, and arthritis. She is almost completely bedridden and has difficulty adhering to her complex medication schedule. After a recent check-up, her e-GFR was calculated as moderate (stage 4) renal insufficiency (28 ml/min/1.73m²). Her medication was adjusted and in the last 6 months, she lost more than 10% of her body weight (8 kilos). Despite this, she still feels exhausted and is completely dependent on her for most activities of daily living.

## D Case 5

### D.1 Description

Sarah is 65 years old, divorced, and has a diagnosis of chronic kidney disease stage 4 (e-GFR = 20ml/min/1.73m²) due to hypertension. She is currently being treated with blood pressure medication, with good results. She has had two hospitalizations in the last year, the first for pyelonephritis and the second for a traffic accident that resulted in a broken leg. She has now fully recovered and is living independently. She has a good appetite and her weight is stable. She was recently made redundant, which prompted her to sell her house and move in search of work.

## E Case 6

### E.1 Description

Alfred is 76 years old, single, and has lived his entire life in a remote valley with a flock of sheep and his dog. Four weeks ago his dog went ‘crazy’ and bit Alfred on his right hand. He did not seek medical advice and the wound became infected and painful, resulting in admission to intensive care for septic shock. After surgical debridement and antibiotics, he began to recover slowly but was bedridden for most of the day. He requires considerable support with activities of daily living. Alfred has lost 4 kg in body weight over the last 6 months. He has been a heavy pipe smoker for at least 40 years and has COPD, with shortness of breath after walking about 50 m

## F Case 7

### F.1 Description

Indra is 86 years old. She is of Surinamese origin and suffers from ischemic heart disease, hypertension, diabetes mellitus type 2, and chronic kidney disease (e-GFR = 46 ml/min/1.73m²). She has been hospitalized twice in the past year, first for pneumonia and later as a result of a fall 6 months later. She lives in her own home and has family help with cooking, cleaning, washing, and dressing. She moves around with a walker but stays in bed or on a chair for most of the day. Her daughter visits her every Tuesday, Friday, and Sunday for at least two hours, and she enjoys it.

## G Case 8

### G.1 Description

Leon is 66 and has been a widower for two years. He misses his wife daily and feels lonely. He has moderate heart failure following a heart attack 2 years ago. He has a history of ulcerative colitis and polymyalgia rheumatica for which he required high doses of steroids, which led to symptoms of diabetes. He has also lost weight and appetite. He has lost 8 kg (*>*10% of his body weight) in the last 6 months, although his appetite has improved with the initiation of a PPI. He spends most of his time in bed, mainly because of pain, and requires extensive nursing care at home from his family and home health nurses. He also has help with housework.

## H Case 9

### H.1 Description

Mohammed is an 89-year-old Turkish man. He has lived with his wife Eslem for 40 years in a stately mansion on the edge of a large city. The couple has four children, with whom they have regular contact. Mohammed was recently diagnosed with left ventricular insufficiency as a result of acute heart failure 20 years ago. He also has Parkinson’s, which makes walking more difficult. He also notices that he has less and less strength in his hands. He has been admitted to the hospital twice in the last 6 months due to cardiac decompensation. He is short of breath with mild exertion and develops edema in his legs during the day. He recently decided to move to a service flat with his wife. Although the new accommodation is quite nice, it was difficult for them to give up their home, where they had lived for more than 40 years. He can take care of himself and help his wife with activities of daily living, share meals with other residents, and take part in excursions with support from the service flats facility.

## I Case 10

### I.1 Description

June is 88 years old. At the age of 80, she divorced her husband Karel. She spent a large part of her life in poverty. This caused her stress. As a result, she drank excessively and smoked an average of a pack of cigarettes per day. June was recently admitted to the hospital with an episode of mild heart failure. She is comfortable at rest but becomes short of breath with moderate exertion such as performing household tasks. This is her second admission for the same problem in the last 10 months. She lives in a residential care facility and before this admission, she spent most of her time in a chair or bed, she maintains a good appetite, she has not lost weight and she can move to the bathroom with the help of the residential care staff.

## J Case 11

### J.1 Description

Thomas is an 80-year-old camera director with moderate dementia, metastatic prostate cancer, type 2 diabetes mellitus, and cirrhosis of the liver. His wife, who is 18 years younger, is his primary caregiver. He has been hospitalized 3 times in the past 9 months. The first time for a urinary tract infection, the second time for pneumonia, and the third time for a fall that broke his nose. He is usually in a good mood. He has a good appetite and his weight is stable. He needs help choosing clothes that are appropriate for the weather, occasion, or season. He sometimes needs help with his care.

## K Case 12

### K.1 Description

Hannah, a 77-year-old divorced woman has moderately advanced Alzheimer’s disease, which has been progressive for the past 4 years. She is a resident of an assisted living facility where she enjoys a good quality of life. Three days ago, on her 76th birthday, she fell and fractured her 6th cervical vertebra. An X-ray showed an unstable fracture and she has not been mobile since the fall. She requires assistance to transfer from her bed to a chair and requires considerable assistance with her care needs. Her son has informed doctors that blood transfusions are not allowed due to her religious beliefs. She has lost her appetite over the past few months and as a result, has lost 2 kg of her body weight in the past month. Sadly, her dog died last week and this has been very upsetting to her.

## L Case 13

### L.1 Description

Isabel is 57 years old, she has a history of smoking of 40 pack years and subsequent severe COPD (Gold D, FEV1 = 52% of predicted) leading to shortness of breath with light activities such as walking 100 m on level ground. Last winter she was admitted to hospital for pneumonia, during which she lost 10 kg. After her discharge, she never recovered to her previous level and is now tired after light housework. In addition, she suffers from ischemic heart disease and unstable angina pectoris, diabetes type 2 with a foot ulcer on the right big toe, and osteoporosis. She can meet most of her daily needs herself, but sometimes she needs help from her husband. Her family lives abroad, from whom she cannot expect support.

## M Case 14

### M.1 Description

Martin, 80 years old and single, suffers from moderate COPD, he has many comorbidities including peripheral vascular disease, ischemic heart disease, and schizophrenia. He has a poor appetite and has lost *>*10% of his body weight in the last six months (5 kilos). He is almost completely bedridden and is capable of limited self-care. He lives in a residential care facility. He has not required hospitalization in the last 18 months.

## N Case 15

### N.1 Description

Mary is a 72-year-old woman diagnosed with moderate COPD. She has recently returned home from her third hospitalization in 9 months for exacerbations of her lung disease. During her last admission, she required non-invasive ventilation and intravenous antibiotics in the acute unit. Last week, her husband Jim was admitted with a stroke and died suddenly. Mary feels depressed and anxious. She is worried that she will not be able to cope without Jim and that her breathing will go haywire again. She can look after herself, in practical terms. She can walk to the local shop which is less than 200 m from her home, but she has to stop halfway to catch her breath. She can cook, dress, and wash herself. Her daughter lives nearby to help with housework and to provide support.
